# N-acetylcysteine mitigates oxidative stress and reduces alveolar bone damage on experimental periodontitis in rats

**DOI:** 10.1007/s00210-026-05421-7

**Published:** 2026-05-14

**Authors:** Ana Paula de Andrade Silva, Erick Alves dos Santos, Deborah Ribeiro Frazão, Vinicius Ruan Neves dos Santos, José Mario Matos-Sousa, Beatriz Risuenho Peinado, Deiweson Souza-Monteiro, Wallacy Watson Pereira Melo, Zuleni Alexandre da Silva, Everton Luiz Pompeu Varela, Jorddy Neves da Cruz, Carina Maciel da Silva-Boghossian, Fabrício Mezzomo Collares, Renata Duarte de Souza-Rodrigues, Rafael Rodrigues Lima

**Affiliations:** 1 Laboratory of Functional and Structural Biology, Institute of Biological Sciences, Federal University of Pará, Augusto Corrêa Street nº 1, Campus Do Guamá, Belém, PA 66075-110 Brazil; 2 Oxidative Stress Research Laboratory (LAPEO), Institute of Biological Sciences (ICB), Federal University of Pará (UFPA), Belém, PA 66075-110 Brazil; 3 Dental Clinic Department, Universidade Federal Do Rio de Janeiro, Rio de Janeiro, RJ 21941-913 Brazil; 4 Dental Materials Laboratory, Department of Conservative Dentistry, School of Dentistry, Federal University of Rio Grande Do Sul, Porto Alegre, RS 90010-150 Brazil

**Keywords:** Alveolar bone loss, Antioxidants, N-Acetylcysteine, Oxidative stress, Periodontitis

## Abstract

This study evaluated the effect of N-acetylcysteine ​​on oxidative biochemistry and bone damage during the development of ligature-induced experimental periodontitis in rats. The rats were divided into three groups (n = 8/group): control, experimental periodontitis (EP), or experimental periodontitis plus daily N-acetylcysteine administration (EP + NA) (100 mg/kg/day). Periodontitis was induced by bilateral silk ligatures on the mandibular first molars for 14 days. The antioxidant capacity of N-acetylcysteine ​​was measured by (2,2-azinobis [3-ethylbenzothiazoline-6-sulfonate] (ABTS• +) and 1,1-dipheny-2-picrylhydrazyl (DPPH•) assays. Gingival samples were collected for analysis of oxidative parameters, while mandibles were used for histopathological evaluations of inflammation and collagen content, as well as for micro-computed tomography assessments of bone quality and vertical bone loss. Data were analyzed by Analysis of Variance (ANOVA), Multivariate Analysis of Variance (MANOVA), and Pearson’s correlation (*p* < 0.05). N-acetylcysteine ​​increased gingival antioxidant capacity and reduced lipid peroxidation compared with the experimental periodontitis group. Histopathological analysis showed that the experimental periodontitis plus daily N-acetylcysteine administration group, compared with the experimental periodontitis group, reduced inflammation, preserving collagen fibers, periodontal ligament, and alveolar bone. Microcomputed tomography analysis revealed improved bone quality and reduced alveolar bone loss in the experimental periodontitis plus daily N-acetylcysteine administration group. These findings suggest that systemic N-acetylcysteine ​​has therapeutic potential in mitigating periodontitis in rats. Further studies are needed to establish its clinical applicability.

**Supplementary Information:**

The online version contains supplementary material available at 10.1007/s00210-026-05421-7.

##  Introduction

Periodontitis is a chronic multifactorial inflammatory disease associated with a dysbiotic biofilm and characterized by the progressive destruction of the tooth-supporting apparatus, including the periodontal ligament and alveolar bone (Papapanou et al. 2018; Tomofuji et al. 2009). Its multifactorial etiology involves the interaction between bacteria present in the oral cavity, the host response, and individual health habits (Sanz et al. 2020). Initially, it manifests clinically as gingivitis, with signs and symptoms such as bleeding during flossing, halitosis, and edema restricted to the marginal gingiva (Slots 2017; Page et al. 1997). As the inflammation extends to deeper tissues, destruction of the periodontal ligament, alveolar bone resorption, and eventually tooth loss occur in susceptible individuals (Trindade et al. 2023).

Periodontitis is considered a major public health problem, with an approximate prevalence of 62% in dentate adults (Papapanou et al. 2018). Worldwide prevalence is estimated to be above 50% (Trindade et al. 2023). In the United States, this condition has already been reported in 40% of the adult population (Eke 2018). The prevalence of severe periodontitis is about 7,4% of the global population (Stødle et al. 2021).

Periodontitis is triggered by gram-negative anaerobic bacteria such as *Porphyromonas gingivalis, Tannerella forsythia, and Treponema denticola*, which are present in deep periodontal pockets (Socransky et al. 1998) (Socransky and Haffajee 2005). These microorganisms release lipopolysaccharides that stimulate macrophages and other inflammatory cells, resulting in the production of pro-inflammatory cytokines such as tumor necrosis factor-alpha (TNF-α), interleukin-1 beta (IL-1β), and prostaglandin E2 (PGE2) (Socransky et al. 1998). These cytokines attract polymorphonuclear neutrophils (PMNs), which generate reactive oxygen species (ROS), leading to an imbalance in antioxidant levels and increasing the expression of receptor activator of nuclear factor kappa-B ligand (RANK-L) in osteoblasts, while also reducing osteoprotegerin (OPG) (Wauquier et al. 2009). This imbalance promotes osteoclast activation, resulting in alveolar bone destruction (Taubman and Kawai 2001; Taubman et al. 2005).

**Fig. 1:**
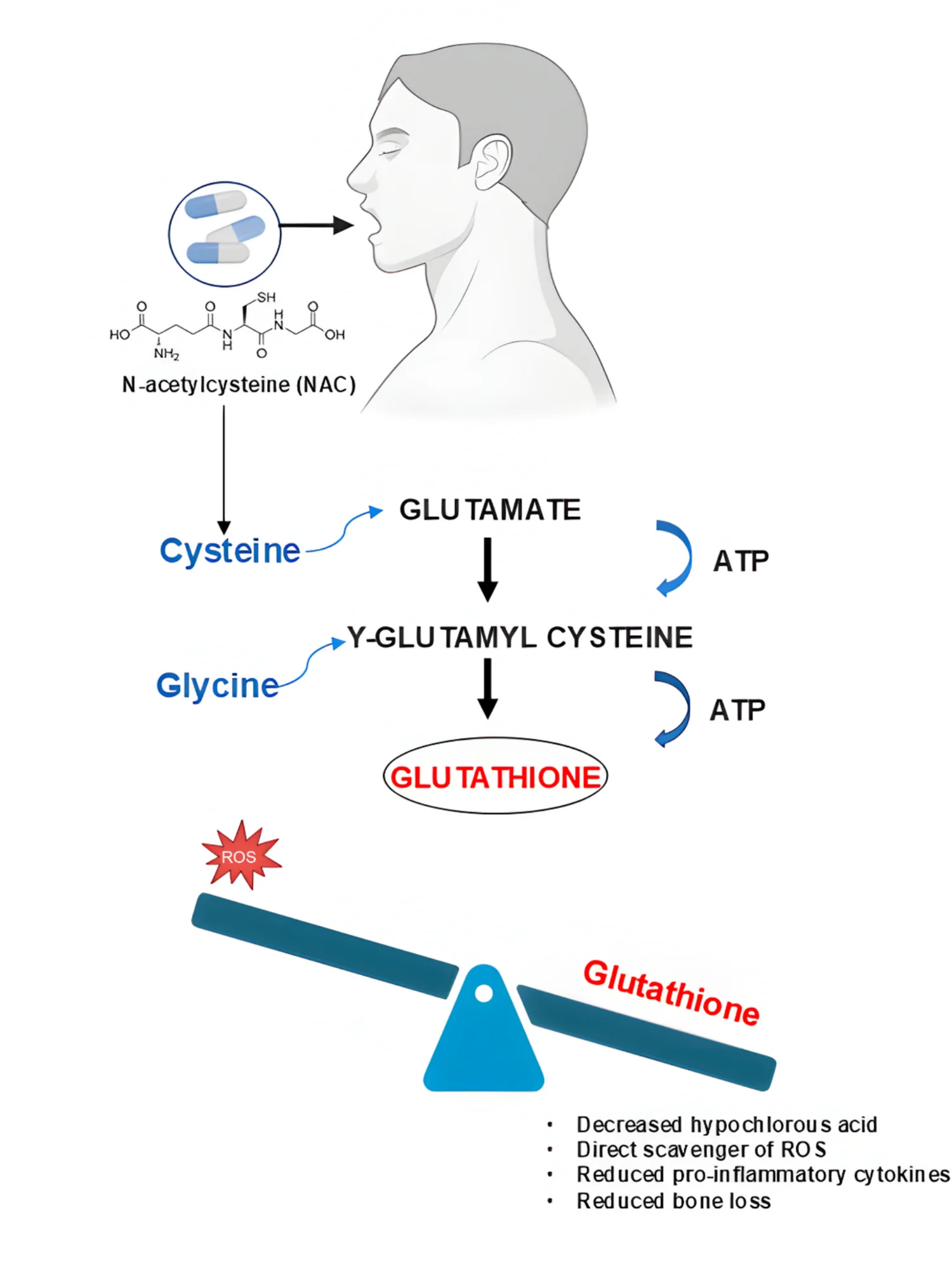
Mechanism of action of N-acetylcysteine in the formation of glutathione. N-acetylcysteine ​​is a precursor of L-cysteine. The increase in L-cysteine leads to the formation of a peptide bond with the amino acid glutamic acid, catalyzed by the enzyme y-glutamylcysteine synthetase. This process results in the formation of y-L-glutamylL-cysteine, which in turn combines with the amino acid glycine through the action of glutathione synthetase, leading to the formation of glutathione. Glutathione plays an important role in reducing the production of hypochlorous acid by polymorphonuclear neutrophils, scavenging reactive oxygen species (ROS), decreasing pro-inflammatory cytokines, and mitigating bone loss. Created with Canva.com

Considering that inflammation and ROS are linked to the development of periodontitis (Sczepanik et al. 2020; Chen et al. 2019), and that studies have identified oxidative stress as an important factor in the progression of the disease, there has been growing interest in the use of antioxidant substances as part of periodontitis treatment (Brock et al. 2004; D’Aiuto et al. 2010). N-acetyl-l-cysteine or N-acetylcysteine ​​(NAC) is recognized as a precursor drug of l-cysteine, when ingested, the body hydrolyzes NAC to produce L-cysteine ​​through a deacetylation process (Samuni et al. 2013; Sjödin et al. 1989; Ueno et al. 2011). Its antioxidant activity is due to its ability to promote the glutathione redox cycle by increasing the amino acid l-cysteine, which in turn binds to the amino acid glutamic acid and glycine, leading to the formation of the tripeptide glutathione (GSH) (Fig. 1) (Ross 1988). Glutathione is one of the main antioxidants responsible for protecting cells against oxidative stress, since GSH acts in the direct sequestration of ROS (Ross 1988).

NAC also acts to combat inflammation triggered by the virulence factor lipopolysaccharide (LPS), released by gram-negative anaerobic bacteria responsible for causing periodontitis (Socranskv et al. 1998; Socransky and Haffajee 2005). Furthermore, it demonstrates beneficial effects in preventing the expression of inflammatory mediators induced by LPS in phagocytic cells and gingival fibroblasts. The anti-inflammatory activity of NAC occurs due to its ability to moderate the interleukin-1 converting enzyme, in which NAC reduces the conversion of interleukin-1β (inactive form of the cytokine) to its active form (Hsu and Wen 2002) Studies have already shown that, in addition to its antioxidant and anti-inflammatory effects, NAC also influences the expression of osteogenic markers such as type I collagen and the microstructure of the alveolar bone. (Yamada et al. 2013; Xi et al. 2022).

Considering that oxidative stress plays a crucial role in periodontitis and that NAC effectively modulates oxidative stress, investigating its effects to minimize disease progression becomes as important as evaluating its effects after the disease onset or during treatment phase. Thus, we hypothesized that NAC ​​would be able to minimize alveolar bone loss and reduce damage to residual bone during the progression of periodontitis. This study aimed to further investigate the role of NAC in modulating oxidative stress and alveolar bone microarchitecture during experimental periodontitis by employing complementary analytical approaches of histological assessments, bone microarchitecture, and oxidative stress to assess both systemic and local parameters.

## Method

### Antioxidant activity of N-acetylcysteine

#### Determination of Trolox equivalent antioxidant capacity (TEAC)

To evaluate the antioxidant capacity of the N-acetylcysteine solution used in our experimental model, the ABTS• + (2,2-azinobis [3-ethylbenzothiazoline-6-sulfonate] diammonium salt radical) and DPPH•(1,1-dipheny-2–picrylhydrazyl) assays were performed using the NAC aqueous solution at 50 mg/mL. The symbol “•” represents a free radical and the symbol “ + ” indicates that the radical is positively charged. This activity was evaluated according to its equivalence to the potent antioxidant Trolox (6-hydroxy-2,5,7,8-tetramethylchromo-2-carboxylic acid) and a synthetic analog of water-soluble vitamin E.

#### ABTS

The evaluation of the elimination of the ABTS• + radical (2,2-azinobis [3-ethylbenzothiazoline-6-sulfonate] diammonium salt radical) was performed using the protocol of Miller et al. (1993) and modified by Re et al. (1999). This method uses a colorimetric technique of the reaction between ABTS (Sigma-Aldrich A1888) and potassium persulfate (K2S2O8; Sigma-Aldrich 60,490), which results in the ABTS• + radical, that reacts with the green/blue colored chromophore. ABTS• + was prepared with 7 mM of this radical and 140 mM of potassium persulfate (K208S2; Sigma-Aldrich 60,490) at room temperature in the dark for 16 h. Subsequently, the solution was diluted with phosphate-buffered saline until obtaining an absorbance of 0.700 (± 0.02) at 734 nm. To standardize the calibration curve, the synthetic antioxidant Trolox was used as a parameter.

To measure the antioxidant capacity, 2.97 mL of the ABTS• + radical solution was transferred to a cuvette, and the absorbance at 734 nm was determined using a Kasvi UV–Vis spectrophotometer. Subsequently, 0.03 mL of the sample was added to the cuvette containing the ABTS• + radical solution, and a second absorbance reading was taken. The results were recorded in mM Trolox equivalents and compared to a Trolox standard (1 mM).

#### DPPH

This method was used to evaluate the potential of NAC to inhibit the 1,1-dipheny-2-picrylhydrazyl (DPPH•) radical, characterized by a color change from violet to yellow or colorless upon the formation of hydrogenated DPPH (M.S. Blois 1958). Initially, the antioxidant capacity of NAC was determined by the absorbance of a 0.1 mM DPPH• solution diluted in ethanol. Then, 0.6 mL of the DPPH solution was mixed with 0.35 mL of distilled water and incubated in a water bath at 37 °C for 30 min. Finally, the absorbance was determined at 517 nm using a Kasvi UV–Vis spectrophotometer. The synthetic antioxidant Trolox was used as a standard solution for the calibration curve. The results were expressed in mM Trolox equivalents.

### Experimental groups

This study was approved by the Animal Care and Use Committee of the Federal University of Pará (UFPA) (CEUA No. 3160221220) and was conducted in accordance with the NIH Guide for the Care and Use of Laboratory Animals. The study also strictly followed the ARRIVE (Animal Research: Reporting of In Vivo Experiments) guidelines (Percie du Sert et al. 2020) (Supplementary material 1).

Based on a previous study using a similar methodology (Dos Santos et al. 2022), the sample size was calculated using G*Power software (version 3.1.9.4) for one-way ANOVA with three groups. Parameters were calculated at an effect size of *f* = 2.209, a Type I error rate of 0.05, and a power of 0.95. This computation indicated a minimum total required sample size of 9 (yielding an actual power of 0.995). Considering a potential loss, the final proposed sample size was 24 rats (8 per group) for this study.

Thus, male rats (*Rattus norvegicus*, *albinus, Wistar*), 90 days old and weighing between 180 and 200 g were selected for this study. The choice of rodents in this age range was made because they are considered adults and musculoskeletal maturity is directly related to age (Ghasemi et al. 2021; Quinn 2005). The animals were provided by the Evandro Chagas Institute (IEC), located in Ananindeua, Brazil. Male rats were chosen over females to avoid hormonal variability associated with the estrous cycle, which could interfere with inflammatory and immune responses (Markou et al. 2009). Animals were randomly allocated to experimental groups using a simple randomization procedure. A random number generator (https://www.random.org/) was used to assign each animal to one of the three groups (n = 8/group): control, experimental periodontitis (EP), or experimental periodontitis plus daily N-acetylcysteine administration (EP + NAC). This procedure was conducted by two of the authors, who were aware of group allocations due to their involvement in the experimental setup. The rats were housed in standard polypropylene cages (dimensions 40 cm × 25 cm × 15 cm) with a maximum of 4 animals per cage to minimize overcrowding. They were provided with controlled food (3 pellets per animal) and water ad libitum. Environmental conditions were maintained on a 12-h light/dark cycle (lights on at 7:00 AM) with a controlled room temperature (25 ± 1 °C) and relative humidity (70 ± 10%). The control group was not subjected to any type of induction and received only distilled water in volumes similar to the animals that were supplemented with NAC; the EP group was subjected to experimental periodontitis and also received only distilled water in volumes similar to those of the animals supplemented with NAC; and the EP + NAC group was subjected to experimental periodontitis and received N-acetyl-l-cysteine ​​supplementation.

### Induction of periodontitis by ligature

To minimize pain and suffering during the experimental procedures, the animals were anesthetized by intraperitoneal injection of a solution containing ketamine hydrochloride (90 mg/kg) and xylazine hydrochloride (9 mg/kg). The depth of anesthesia was confirmed by the loss of corneal reflexes and the absence of response to finger pinch. The animals were then positioned on an immobilization table, and the oral cavity was kept open to allow insertion of a silk ligature around the cervical region of the right and left mandibular first molars for 14 days to induce experimental periodontitis. Previous studies have demonstrated that this induction model and the experimental time used are capable of effectively simulating the bone loss observed in humans (Frazão et al. 2020; Dos Santos et al. 2022; Vargas-Sanchez et al. 2017). Throughout the 14-day experimental period, the animals were monitored daily for signs of pain, distress, or adverse events, such as weight loss, reduced mobility, or changes in feeding behavior. No adverse events were observed during the study. Humane outcomes were predefined as severe weight loss (> 20% of body weight), prolonged lethargy, or inability to access food or water. If any animals had met these criteria, they would have been euthanized immediately; however, no animals met these criteria.

### N-acetylcysteine supplementation

Animals assigned to the EP + NAC group received N-acetylcysteine supplementation at a dosage of 100 mg/kg/day in a 50 mg/mL aqueous solution diluted with distilled water, in accordance with the dosage previously used in another study (Miranda Filho et al. 2021). Supplementation with NAC via orointragastric gavage was initiated 24 h after ligation placement and continued daily for 14 days. To ensure accurate dosing adjustments, the animals were weighed every 7 days throughout the 2-week experiment period. Animals in the other groups received an equivalent volume of distilled water, by orointragastric gavage, over the same period as animals in the EP + NAC group.

### Euthanasia and sample collection

**Fig. 2:**
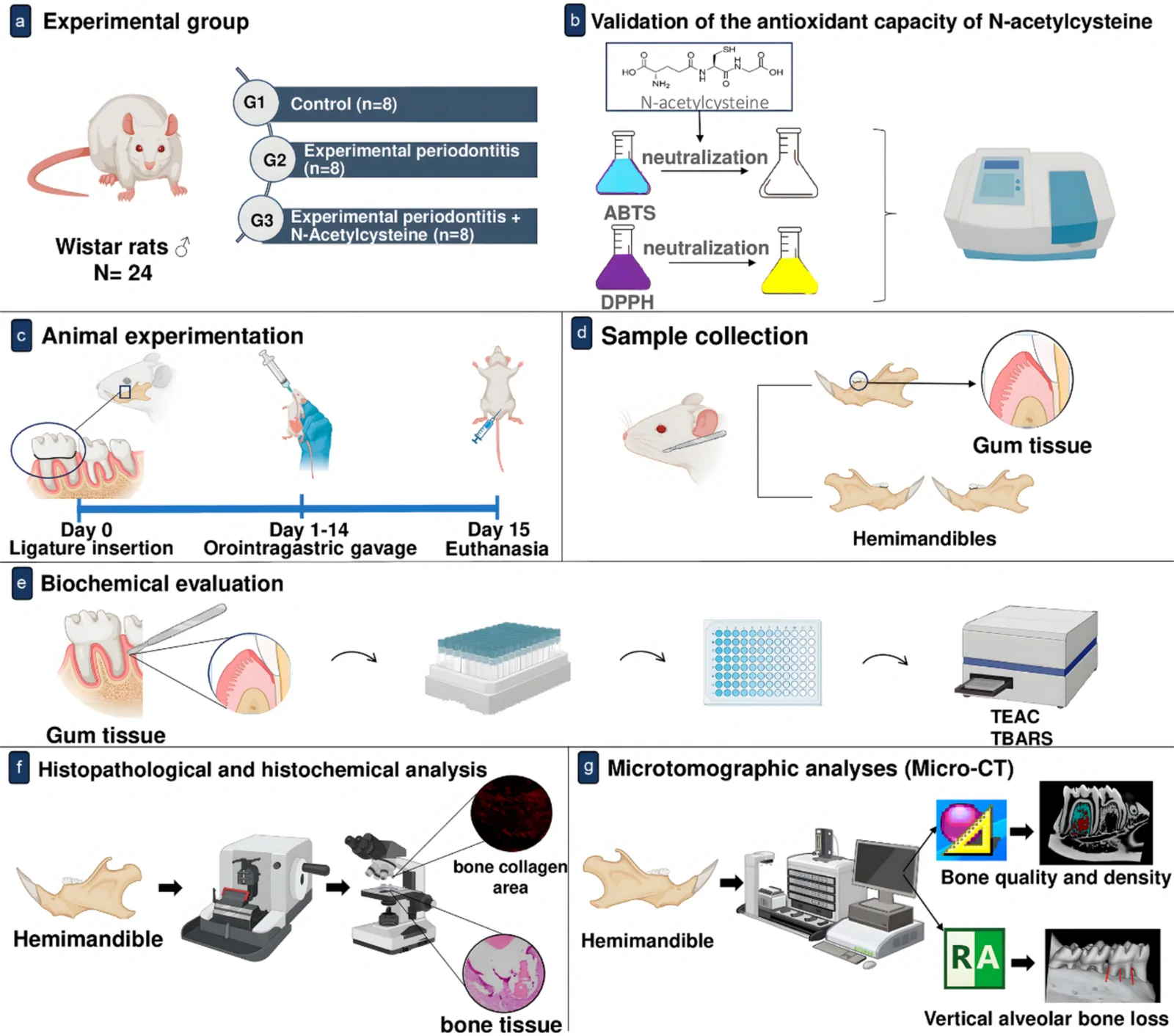
Experimental design.** a**- Randomized experimental groups. **b**- Validation of the antioxidant capacity of the N-acetylcysteine ​​solution used. **c**- Schedule of the experimental period: day 0 was characterized by the insertion of ligatures bilaterally in the first lower molar. After this stage, from day 1 to day 14, the animals in the control and experimental periodontitis groups received distilled water, and the animals in the periodontitis + NAC group were supplemented with N-acetylcysteine. On the 15th day, the animals in all groups were euthanized. **d**- Representation of the gingiva and mandible samples collected after euthanasia. **e**- Representation of the biochemical analysis with gingiva samples for local oxidative evaluation. **f**- Representation of the histopathological and histochemical analysis, in which the mandibles were processed for histological analysis of the bone tissue and collagen area. **g**- Representation of the micro-computed tomography (Micro-CT) analysis of the mandible samples for measuring vertical bone loss and bone quality. Created with BioRender.com

On the 15th day, 24 h after the last administration of N-acetyl-L-cysteine, the animals were anesthetized intraperitoneally with a solution of ketamine hydrochloride (90 mg/kg) and xylazine hydrochloride (9 mg/kg) to ensure they were fully unconscious and free from pain during tissue collection. The depth of anesthesia was confirmed by the absence of corneal reflexes and response to toe pinch. The gingival tissue around the first two lower molars was collected through an incision with a scalpel and a slight detachment, taking care to minimize tissue damage. The delimitation of the collected gingival fragment was performed following the anatomical contours from the free gingival margin to part of the attached gingiva, taking anatomical orientation into account. The tissue was immediately stored at −80 °C for subsequent biochemical analyses. Blood samples were also collected through cardiac puncture and transferred to K3 tubes containing heparin and then centrifuged for 10 min at 3000 rpm, and the obtained plasma was stored at −80 °C. The animals were then perfused with 0.9% heparinized saline and 4% formaldehyde introduced through the left ventricle of the heart. After this, the mandible samples were collected and divided in half. One-half of the mandible was used for evaluation in micro-computed tomography (Micro-CT), while the other half was used for histopathological and histochemical analysis. To minimize bias during outcome assessment and data analysis, the researchers performing histological, biochemical, and micro-CT analyses were blinded to group assignments. The methodological scheme and the analyses performed are shown in Fig. 2.

### Oxidative biochemical analysis

#### Gingival biochemical analysis

To analyze local oxidative parameters, the animals' gums were collected bilaterally and submerged in saline solution, then frozen with liquid nitrogen and stored at −80 °C. These samples were thawed and resuspended in Tris-20 mM HCl, pH 7.4, at 4 °C, by sonic disintegration (approximate concentration of 1 g/mL). The supernatant was stored at −80 °C until processing (Dos Santos, V. R. N 2022). The amount of proteins in the samples was measured to correct the biochemical data using the method of Bradford (1976). Then, the TEAC and LPO assays were performed since even though NAC does not act directly on the inhibition of TEAC and LPO, previous studies have shown that its indirect antioxidant effects may influence the results of these tests (Skrzydlewsk aand Farbiszewski 1999; Muñoz-Sánchez et al. 2023).

##### Trolox equivalent antioxidant capacity (TEAC

To determine TEAC levels, the methodology ecommended by Miller et al. (1993) and adapted by Re (1999) was used. The gingival samples were placed in the ABTS• + radical solution, followed by a calorimetric reading. The results were expressed in mM/ml for plasma and mM/g of protein for the tissue samples that underwent hemolysis.

##### Lipid peroxidation (LPO)

Malondialdehyde (MDA) levels were measured as an indicator for LPO (Esterbauer and Cheeseman 1990) levels. First, the gingival samples were homogenized using sonication, followed by centrifugation of the supernatants at 3512 × g for 10 min at 4 °C. Then, the supernatants with MDA solutions were incubated with a 1:4 mixture of methanesulfonic acid solution and 10.3 mM N-methyl-2-phenylindole (diluted in methanol at 1:3 ratio) in a water bath at 45 °C for 40 min. Absorbance was measured using a spectrophotometer at λ = 570 nm. The results were expressed in nanomoles per microgram (nmol/µg) of protein.

### Histopathological and histochemical analysis

The hemimandibles were post-fixed in 4% formalin solution for 24 h, then rinsed in running water for 2 h and stored in 10% ethylenediaminetetraacetic acid (EDTA) solution for 180 days. The EDTA solution was changed weekly until histological processing was performed. After decalcification, the samples were washed in running water and dehydrated in increasing concentrations of ethanol, diaphanized in xylene and embedded in paraffin. Finally, the paraffin blocks obtained were cut in a Leica RM 2045 microtome (Leica Microsystems, Nussloch, Germany) at a thickness of 5 µm in the transversal direction and the tissues obtained were stained with hematoxylin–eosin (HE) and PicroSirius Red.

The slides that underwent HE staining were sent for optical microscopy investigation of the tissues of the hemimandibles. The analysis consisted of a qualitative comparison between the experimental groups of the region of the bone crest of the alveolar process between the molar roots. The images of the slides stained with HE were captured with a color digital camera (C-mount, DS-Fi3, Nikon, Tokyo, Japan) connected to an optical microscope (Eclipse Ci-L, Nikon, Tokyo, Japan).

Slides stained with PicroSirius Red were used to estimate the collagen area in each sample, using ImageJ software (NIMH, NIH, Bethesda, MD, United States) along with polarized light microscopy to calculate the arithmetic mean of the threshold percentages from five fields/sections (Junqueira et al. 1978; Matos-Sousa et al. 2024). The collagen area was recorded as a percentage (%). Sections were deparaffinized in xylene, dehydrated in an ethanol gradient, and then stained with 0.1% PicroSirius Red for 60min, and rinsed with hydrochloric acid for 2 min (Yu et al. 2019). Sections were observed using light microscopy and analyzed with the aid of a polarizer attached to the microscope. Images for all three analyses were taken with a color digital camera (Cyber Shot DSC W-230, 4X optical zoom, Sony, Tokyo, Japan) attached to a microscope (1.5X, Eclipse E200, Nikon, Tokyo, Japan) at magnifications of 10X and 40X.

### Micro-computed tomography (Micro-CT) analysis

The hemimandibles were mounted on a rotating platform inside the device and the images were taken in a 360º rotation with an intensity of 70 kV and 100 mA, being reconstructed in the inspeXio SMX-90CT software program (Shimadzu Corp., Kyoto, Japan) with a voxel size of 10 µm in images at a resolution of 1024 × 1024 and 14 µm in thickness.

Bone loss was assessed in terms of height, using the RadiAnt DICOM Viewer 5.0.1 (Medixant, Poznan, Poland) for three-dimensional reconstruction of the hemimandibles. The three-dimensional models were positioned in a standard position, allowing visualization of the buccal and lingual surfaces of the teeth. Thus, vertical bone loss was identified by measuring the distance between the cementoenamel junction and the alveolar bone crest at six points on the first mandibular molar (mesiobuccal, buccal, distobuccal, distolingual, lingual and mesiolingual), with calculation of the mean of these regions.

The CTAn software (V1.15.4.0; Bruker, Kontich, Belgium) was used to generate microtomographic images and verify the alveolar bone quality. For this purpose, the region of interest (ROI) was determined in a set of 60 images of the alveolar bone region involving the roots of the first molar, i.e., in the interradicular region. The software adjusted the applicable threshold according to the guidelines provided by the manufacturer, with a limit (31–71) applied to the segmentation of the different shades of gray present in the image. We performed a two-dimensional reconstruction analysis of the hemimandibles using qualitative, subjective evaluation of the images. This method involved visual comparisons between groups to determine bone structure integrity. Additionally, quantitative parameters were assessed by calculating the bone volume-to-tissue volume ratio (BV/TV), number of trabeculae (Tb.N) and measuring trabecular thickness (Tb.Th). All bone microarchitecture analyses were performed by a qualified dental surgeon with expertise in micro-computed tomography analysis.

### Statistical analysis

All analyses were performed using Graphpad Prism 9.0 software. Data from the three experimental groups were tabulated and the Shapiro–Wilk test was applied to test normality. For parametric data, one-way Analysis of Variance (ANOVA) with Tukey’s post-test was applied. The significance level adopted was *p* < 0.05. Next, Pearson’s correlation test was performed to verify the correlation between the parametric data of TEAC and LPO, as well as local oxidative biochemistry. After that, a Multivariate Analysis of Variance (MANOVA) was performed to test the hypothesis that the means of the dependent variables differed between the experimental groups. The Pillai's Trace, Wilks Lambda, Hotelling’s Trace and Roy’s Largest Root tests were applied to assess the significance of the differences between the groups, considering a value of *p* < 0.05 as statistically significant (The jamovi project 2021; R Core Team 2021; Jarek 2012). These data were evaluated using Jamovi software version 2.6.13.

## Results

### N-acetylcysteine was able to neutralize ABTS•+ and DPPH• radicals 

The analysis of the ABTS• + and DPPH• radicals in the NAC solution used in the present study indicated that NAC has a high antioxidant capacity. In the results of the ABTS• + assay, NAC exhibited a moderate antioxidant capacity, when compared to the Trolox standard. In contrast, the DPPH• assay demonstrated that the NAC used in this study has a high antioxidant capacity, similar to that of the Trolox standard. The results are presented in Table 1.

**Table 1:** Representation of the average of 2,2-diphenyl-1-picrylhydrazyl (DPPH•) and 3-ethylbenzothiazoline-6-sulfonic acid (ABTS• +) free radicals neutralized by N–acetylcysteine

N–Acetylcysteine		
	Mean (mM)	SD
DPPH•	0.910	0.027
ABTS•^+^	0.320	0.004

### Biochemical analysis of gingival tissue

#### N-acetylcysteine elevated TEAC levels

**Fig. 3:**
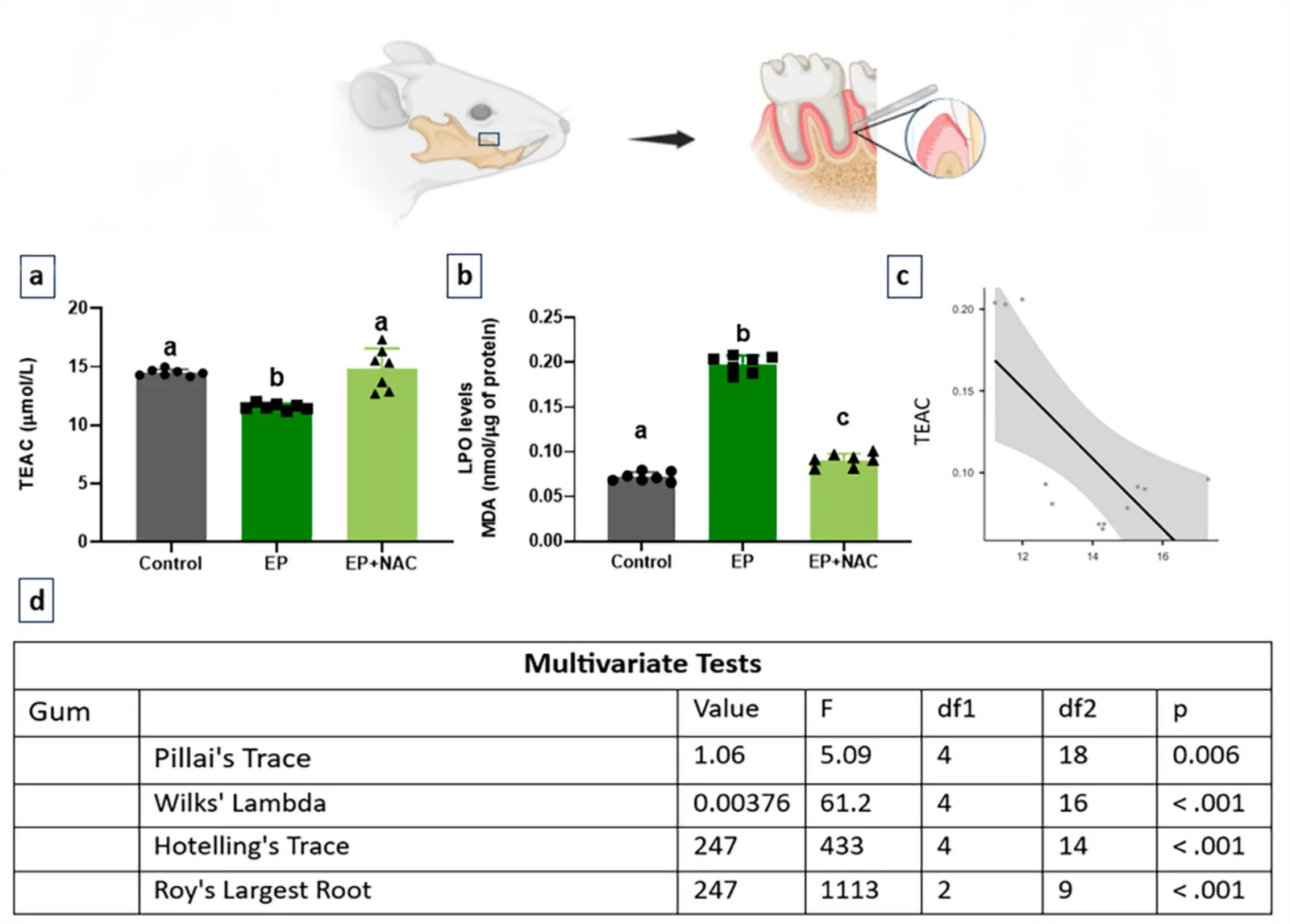
Levels of local antioxidant biomarkers in the gingival tissue of rats subjected or not to experimental periodontitis and systemic supplementation of N-acetylcysteine (100 mg/kg/day). (**a**): Total Antioxidant Capacity Equivalent to Trolox (µmol\L); (**b**): Lipid peroxidation (nmol\µg). One-way ANOVA with Tukey’s post-test was used (*p* < 0.05). (**c**): TEAC-LPO correlation graph. (**d**): Table with the results of the Pillai's Trace, Wilks Lambda, Hotelling's Trace and Roy's Largest Root multivariate tests

The analysis of the antioxidant capacity equivalent to Trolox—TEAC (Fig. 3A) demonstrated that the EP + NAC group (14.79 ± 0.6697 µmol/L) presented statistically higher TEAC levels than the EP group (11.58 ± 0.09775 µmol/L; *p* < 0.0001); while EP + NAC group and control group (14.50 ± 0.1057 µmol/L; *p* = 0.8671) had similar TEAC levels.

#### N-acetylcysteine reduced lipid peroxidation levels compared to the experimental periodontitis group

In the lipid peroxidation (LPO) analysis (Fig. 3B), it was observed that the EP group presented higher levels compared to the control group (0.1982 ± 0.003580 nmol/μg vs 0.07219 ± 0.001990 nmol/μg; *p* < 0.0001). On the other hand, the EP + NAC group showed a significant reduction in LPO levels compared to the EP group (0.09067 ± 0.002777 nmol/μg vs 0.1982 ± 0.003580 nmol/μg; *p* < 0.0001), although it still had higher LPO levels than the controls (0.09067 ± 0.002777 nmol/μg vs 0.07219 ± 0.001990 nmol/μg; *p* = 0.0007).

The Pearson correlation test and MANOVA revealed a negative relationship between LPO and TEAC (*r*: −0.696, *p* = 0.012) and significant differences in biochemical tests (*p* < 0.001). Multivariate tests showed notable group differences: Pillai’s Trace = 1.06, *p* = 0.006; Wilks Lambda = 0.00376, *p* < 0.001; Hotelling’s Trace = 247, *p* < 0.001; Roy’s Largest Root = 247, *p* < 0.001. These results indicate statistically significant differences among groups when considering the dependent variables together (Fig. 3C and D).

### Histopathological analysis

**Fig. 4:**
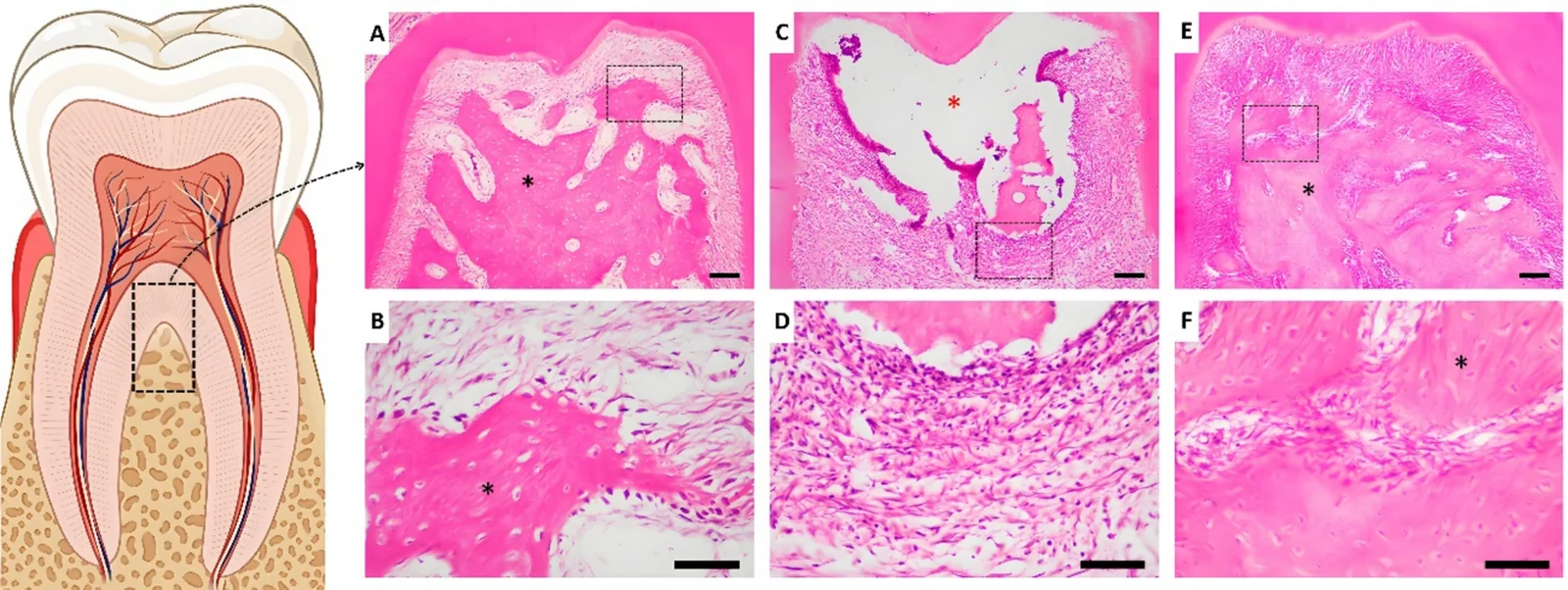
Macroscopic histopathology of the periodontium of rats with experimental periodontitis that received supplementation with N-acetylcysteine ​​(100 mg/kg/day) for 14 days. The illustration of the human tooth is used representatively to indicate the region of anatomical correlation in the analyzed rats. The letters **a** and **b** represent the control group; **c** and **d**- experimental periodontitis group; **e** and **f**—experimental periodontitis group + N-acetylcysteine. 10 × and 40 × magnification. Scale bar 20 µm

Histopathological analysis demonstrated that NAC effectively mitigated tissue alterations caused by periodontitis (Fig. 4). In the control group, the tissues were healthy, in physiological conditions, with clear preservation of the alveolar bone, periodontal ligament, and root surface integrity (Fig. 4A). Osteocytes in the alveolar bone were well-preserved, and the periodontal ligament showed typical fibroblasts and ample collagen fibers, indicating low tissue stress (Fig. 4B). In contrast, the EP group showed significant pathological changes, with evident tissue discontinuity and pronounced alveolar bone and root surface resorption. This was accompanied by a severe inflammatory infiltrate, predominantly lymphocytes, and plasma cells, and a notable loss of collagen fibers, highlighting the destructive impact of periodontitis, there is also neovascularization and granulation tissue (Fig. 4C, D). Remarkably, in the EP + NAC group, these pathological changes were significantly reduced. There was a clear preservation of both the periodontal ligament and alveolar bone, with a marked reduction in the inflammatory infiltrate, suggesting that NAC treatment promotes tissue repair and helps maintain a state closer to physiological health (Fig. 4E and F).

### PicroSirius red analysis

**Fig. 5:**
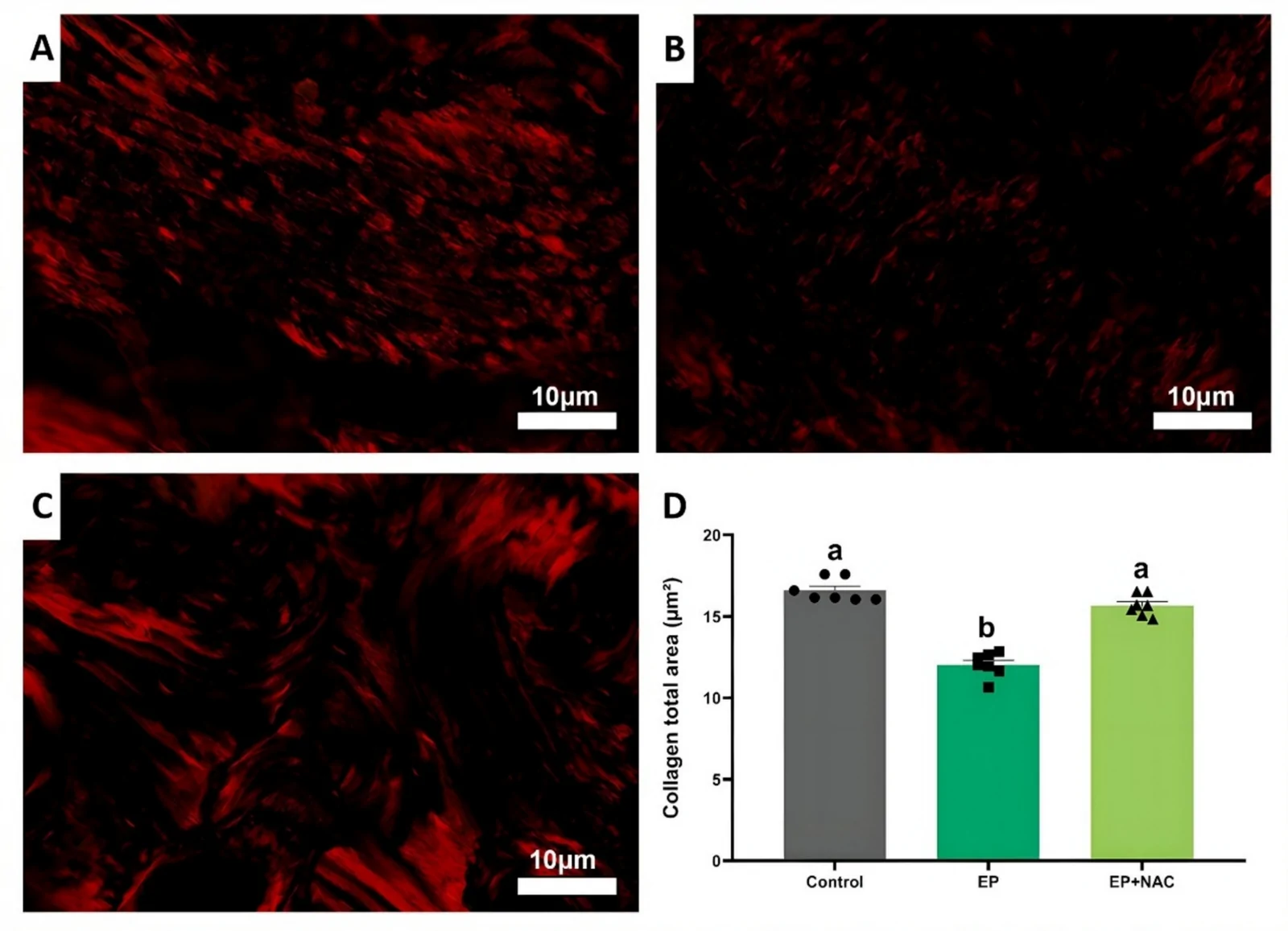
Effects of N-acetylcysteine ​​supplementation (100 mg/kg/day) for 14 days on collagen fibers of alveolar bone of rats with periodontitis. Photomicrographs **a**, **b**, **c** represents the area of ​​PicroSirius Red staining of the control group, experimental periodontitis group and experimental periodontitis supplemented with N-acetylcysteine, respectively. In **d**, the percentage of bone collagen area (%) is presented. Scale bar: 10 μm. The ANOVA test followed by Tukey’s post-hoc test (*p* < 0.05) was used. The images were captured using polarized light microscopy

In the analysis of the collagen fiber area (Fig. 5),was able to mitigate the damage caused by periodontitis, since the EP + NAC group (Fig. 5C) presented a larger collagen area when compared to the EP group (Fig. 5B) (12.03 ± 0.2803 µm^2^; 15.66 ± 0.2504 µm^2^; *p* < 0.0001). Furthermore, it was possible to observe that the EP + NAC group ​​presented a collagen area equivalent to the control group (16.60 ± 0.2645 µm^2^; 15.66 ± 0.2504 µm^2^; *p* = 0.0557) (Fig. 5A).

### Micro-computed tomographic analysis

**Fig. 6:**
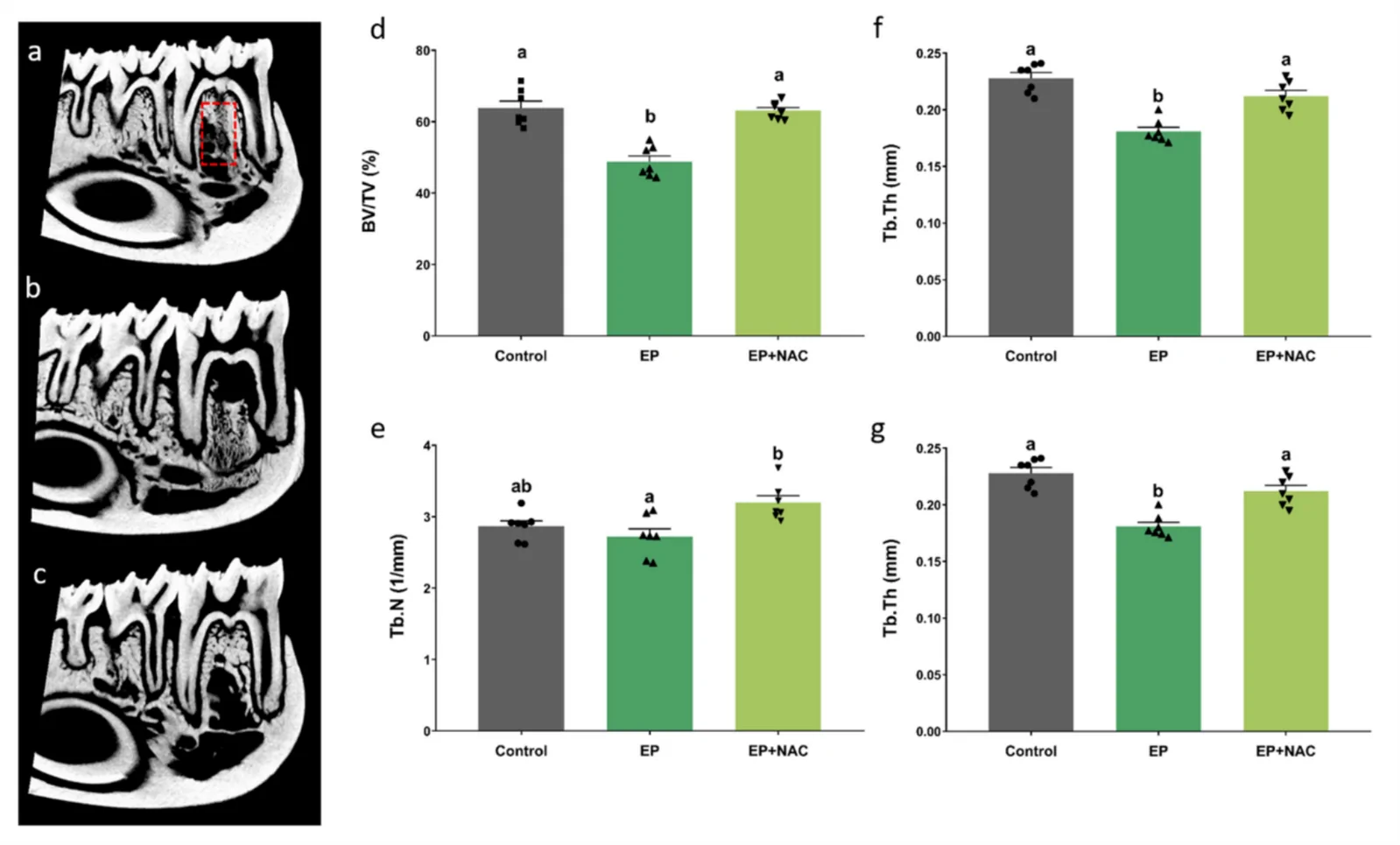
Microcomputed tomographic analysis of bone quality parameters: (**a**) Representative sagittal section of the hemimandible in the region of the first lower molar for the control group (**b**), the experimental periodontitis group (**c**) and the experimental periodontitis + N-acetylcysteine ​​group. The area marked in red indicates the region of interest for measuring the quality of the alveolar bone in the interradicular region. Quantitative analyses include (**d**) percentage of bone volume (BV/TV, %), (**e**) number of trabeculae (Tb.N; 1/mm), (**f**, **g**) trabecular thickness (Tb.Th, mm). Groups with identical letters do not show significant differences (*p* > 0.05). Statistical comparisons were performed using ANOVA followed by Tukey's post hoc test

The 2D reconstruction of the hemimandibles (Fig. 6a–c) demonstrated the region of interest for measuring bone quality. The quantification of bone quality parameters revealed a significant reduction in the bone volume-to-tissue volume ratio (BV/TV) in the EP group (48.80 ± 1.612%) compared to the control group (63.87 ± 1.916; *p* < 0.0001; Fig. 6d). In contrast, the EP + NAC group presented a significantly higher BV/TV (63.10 ± 0.9034%; *p* < 0.0001) compared to the EP group. Furthermore, NAC supplementation preserved bone volume at levels comparable to those observed in the control group (*p* = 0.9329).

The EP + NAC group ​​had a higher number of trabeculae (Tb.N) compared to the experimental periodontitis group (3.198 ± 0.09648 1/mm; 2.723 ± 0.1090 1/mm; p = 0.0060; Fig. 6e). Both the control group and the EP + NAC group ​​had greater trabecular number when compared to the EP group (2.869 ± 0.07425 mm and 3.198 ± 0.09648 mm vs. 2.723 ± 0.1090 mm; *p* < 0.05; Fig. 6e). There was no statistically significant difference found between the EP + NAC group and the control group (*p* = 0.0591).

Moreover, a significant reduction in trabecular thickness (Tb.Th) was observed in the EP group (0.1808 ± 0.003798 mm) compared to the control group (0.2280 ± 0.004801 mm; *p* < 0.0001; Fig. 6f, g). Furthermore, the experimental periodontitis group exhibited significantly lower Tb.Th compared to the N-acetylcysteine group (0.2121 ± 0.004983 mm; *p* = 0.0003), while no statistically significant difference was found when compared to the control group (*p* = 0.0599).

### N-acetylcysteine supplementation provided a reduction in alveolar bone loss caused by experimental periodontitis

**Fig. 7:**
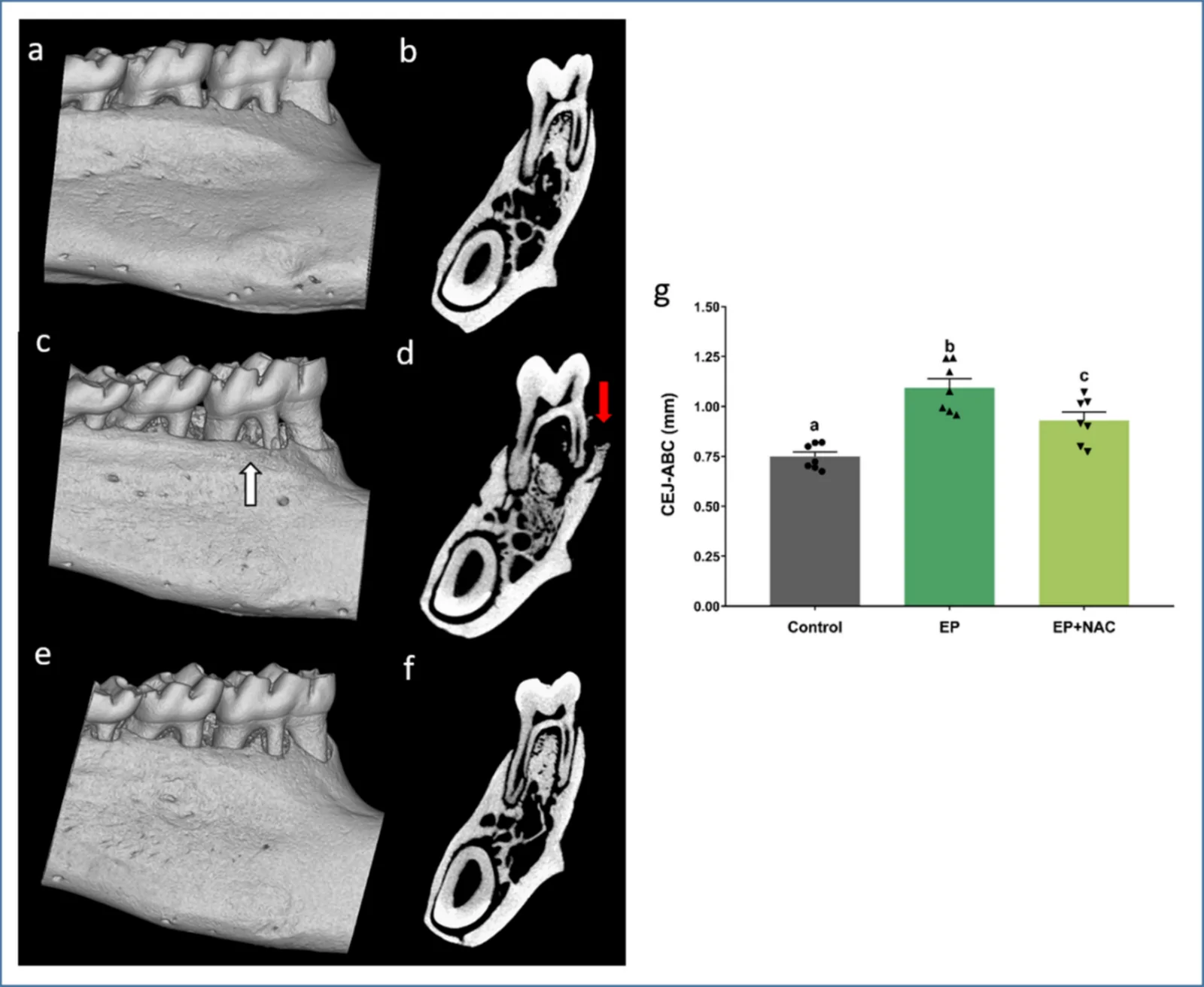
2D and 3D reconstruction of the hemimandibles to evaluate vertical bone loss in rats with experimental periodontitis that received N-acetylcysteine ​​supplementation (100 mg/kg/day) for 14 days. The white arrow shows the vertical levels analyzed to measure alveolar bone loss in 3D and the red color arrow identifies the alveolar bone crest level in 2D. (**a**, **b**) Control group (**c**, **d**) Experimental periodontitis and (**e**, **f**) Experimental periodontitis + N-acetylcysteine. (g) Distance from the cementoenamel junction (CEJ) to the alveolar bone crest (ABC) in mm for all experimental groups. The results are expressed in mm of the mean ± standard error of the mean. Different letters indicate statistically significant differences. One-way ANOVA with Tukey’s post-test was used (*p* < 0.05).

The 3D and 2D reconstruction of the hemimandibles exhibited intact bony crests and alveolar bone structures in the control groups (Fig. 7a, b). In contrast, the EP group (Fig. 7c, d) showed significant crestal bone resorption and notable alveolar bone loss represented by the red arrow. On the other hand, the EP + NAC group (Fig. 7e, f) showed preservation of the bone crest and alveolar bone.

When performing the quantitative analysis, the EP group presented significantly greater alveolar bone loss compared to the control group (1.093 ± 0.04706 mm vs 0.7479 ± 0.02369 mm; *p* < 0.0001; Fig. 7g). In contrast, the EP + NAC group showed a remarkable reduction in alveolar bone damage compared with the EP group (0.9288 ± 0.04321; *p* = 0.0221; Fig. 7g). This result is evidenced by the shorter distance between the cementoenamel junction and the alveolar bone crest (CEJ-ABC).

## Discussion

In this study, we investigated, for the first time, the effects of NAC on the modulation of gingival oxidative biochemistry and on the protection of alveolar bone microstructure and morphology during the development experimentally induced periodontitis. Our results demonstrated that NAC not only reversed the loss of antioxidant capacity but also mitigated lipid peroxidation associated with periodontal disease progression. Furthermore, NAC administration reduced the loss of periodontal tissue integrity, minimizing the inflammatory burden and preserving the total collagen area. Micro-CT analysis showed that there was a significant increase in the ratio between bone volume, and total tissue volume and number of trabeculae and in trabecular thickness. These effects culminated in a reduction in vertical loss in alveolar bone. Thus, our findings suggest a potential antioxidant and protective effect of NAC on periodontal tissues during disease progression.

The literature indicates that the use of NAC ​​is safe and effective at both higher and lower doses. One study demonstrated that doses of 25 mg/kg/day were useful in improving dyslipidemia parameters (Kaga et al. 2018). Another study demonstrated that doses of 600 mg/kg/day reduce lipid peroxidation and increase systemic total antioxidant capacity in diabetic rats (Argaev Frenkel et al. 2018). We chose to use NAC supplementation at a dosage of 100 mg/kg/day, following the positive results observed in a previous study that analyzed oral cavity organs, including the submandibular salivary glands of diabetic rats (Miranda Filho et al. 2021).

In the specific context of gingival oxidative biochemistry, we found that NAC increased the total antioxidant capacity and reduced lipid peroxidation compared to non-supplemented groups. This demonstrates that, despite being administered systemically, NAC led to local antioxidant effects. Furthermore, Pearson correlation and MANOVA analysis corroborated these findings by indicating that high lipid peroxidation levels in the periodontitis-only group are associated with low total antioxidant capacity levels, thus demonstrating an oxidative biochemical imbalance in the region (Juczunk et al. 2007). However, according to our results, the experimental periodontitis group supplemented with NAC presented low lipid peroxidation levels associated with high total antioxidant capacity levels. Thus, we can suggest that the supplementation with NAC resulted in significant effects in the modulation of local oxidative stress. In periodontitis, several inflammatory cells produce ROS as part of the body’s response to microbial aggression. Among the primary sources are polymorphonuclear neutrophils (PMNs) and fibroblasts, which play important roles in the development and progression of periodontal tissue destruction (Dos Santos et al. 2022). The interaction between inflammation and oxidative stress reflects interconnected mechanisms of the immune response, which are also implicated in the onset and progression of several diseases (Hajishengallis, G 2020).

Previous studies have suggested several mechanisms through which NAC may influence bone and inflammatory processes. First of all, NAC acts as a precursor of glutathione and as a direct scavenger of reactive oxygen species (ROS), thereby contributing to the restoration of redox balance in inflammatory conditions (Alksdasi et al. 2017). Experimental evidence also indicates that NAC may modulate pathways involved in bone metabolism, including the stimulation of alkaline phosphatase activity, the upregulation of genes associated with osteoblast differentiation, and the increased synthesis of bone morphogenetic proteins (Alam et al. 2014; Sun et al. 2023). In addition, NAC has been reported to interfere with inflammatory signaling by suppressing mediators such as RhoA and COX-2 and by reducing the production of pro-inflammatory cytokines including TNF-α, IL-6, and IL-1β. These effects have been linked to the modulation of signaling pathways such as NF-κB, JNK, ERK, and MAPK, which are activated in response to bacterial lipopolysaccharides during periodontal inflammation (Sun et al. 2023).

Although these molecular pathways were not directly evaluated in the present study, they may help explain the biological effects observed here. Oxidative stress and inflammation are known to disrupt bone metabolism by impairing osteoblast differentiation and promoting osteoclast activity, processes frequently associated with alterations in the RANKL/OPG balance (Iantomasi et al. 2023; Kimball et al. 2021). By mitigating oxidative stress, NAC may indirectly contribute to restoring the balance between bone resorption and bone formation, which could partly explain the preservation of alveolar bone microarchitecture observed in our experimental model (Kimball et al. 2021; Maruyama et al. 2020).

These local and systemic findings of the antioxidant effects of NAC in periodontitis may justify those found in histopathological analysis, which in turn showed that the supplemented group had a lower concentration of inflammatory infiltrate when compared to the experimental periodontitis group. Moreover, NAC supplemented group also showed maintenance of osteocytes, which are dynamic and multifunctional cells. Osteocytes integrate mechanical and hormonal signals, thereby thus coordinating the differentiation and function of osteoclasts and osteoblasts, making them essential for maintaining bone homeostasis (Cui et al. 2022; Delgado-Calle and Bellido 2022). In addition, the histopathological analysis also shows that the treatment with NAC also promoted the maintenance of fibroblasts population, when compared to the non-supplemented group. The results obtained corroborate the analysis of collagen content, which revealed a significant loss of these fibers in the experimental periodontitis group as a result of the inflammatory process. This loss negatively impacts the quality of the bone matrix (Matos-Sousa et al. 2024).

The extracellular matrix, composed of collagens, glycoproteins and proteoglycans in the interstitial fluid medium, plays a crucial role in regulating cellular behavior, including adhesion, proliferation and the cell cycle (Zardeneta et al. 2000). Studies indicate that oxidative stress can destabilize this matrix, affecting its components, such as collagen, which can have its synthesis modulated, its triple helix structure unfolded, in addition to undergoing fibronectin fragmentation and fibrinogen oxidation (Zardeneta et al. 2000; Rees et al. 2008). These imbalances help to explain the histochemical findings, which demonstrated the reduction of collagen fibers caused by periodontitis. Conversely, the preservation of collagen fibers was observed in the group supplemented with NAC, which had similar characteristics to those of the control group. These results suggest that NAC was effective in attenuating the effects of periodontitis on collagen fibers of the extracellular matrix, due to its antioxidant action (Ueno et al. 2011; Ross 1988).

By attenuating systemic oxidative and inflammatory processes, NAC alleviated bone loss and improved the alveolar bone quality, evidenced by current micro-computed tomographic analysis, thereby promoting the restoration of bone homeostasis. Having in mind current and previous findings, NAC shows promising potential in bone tissue engineering and regeneration. For example, it has enhanced the osteogenic potential and immune regulatory function of cells (Zheng et al. 2024). NAC can also increase alkaline phosphatase activity, promote mineralized matrix formation, and upregulate bone-related gene markers in osteoblastic cultures (Yamada et al. 2013).

We observed that NAC preserved the bone volume-to-tissue volume ratio (BV/TV), indicating that in the region of alveolar bone remaining after bone loss, a substantial amount of bone volume was maintained within the selected region of interest. This preservation may be attributed to the maintenance of trabecular thickness. Notably, in the periodontitis group, trabecular thickness was significantly reduced, rendering the bone more fragile and potentially porous. Interestingly, NAC supplementation led to the maintenance of the trabecular thickness at levels comparable to the control group. In addition to enhancing bone quality, the supplemented NAC group maintained the distance between the cementum-enamel junction and the alveolar bone crest. This suggests that a dosage of 100 mg/kg/day for 14 days could effectively prevent bone resorption during the progression of periodontitis in rats. This is in accordance with another study, in which NAC accelerated bone regeneration in rat femur defects (Yamada et al. 2013). Additionally, it was demonstrated that mice fed a high-fat diet and supplemented with NAC had increased bone mass by elevating glutathione status and decreasing osteoclast differentiation (Cao and Picklo 2014). These findings suggest that NAC could be valuable for enhancing bone regeneration and maintaining bone health.

Despite the promising findings, this study has some limitations. First, our results are based on an animal model, which may not directly translate into clinical applications in humans. Further research is needed to evaluate the efficacy and safety of NAC in clinical settings, considering the differences in physiology and responses between species. Also, this study assessed the outcomes of a single dosage (100 mg/kg/day) and treatment duration (14 days). Furthermore, it is important to note that NAC administration began shortly after ligature placement; therefore, the present study evaluated its modulatory effects during disease development rather than its therapeutic efficacy in established periodontitis. Future studies should explore the effects of varying dosages and longer treatment periods to better understand the dose–response relationship and the potential long-term benefits or risks of N-acetylcysteine. Additionally, research should investigate potential adverse or toxic effects associated with prolonged use of this substance, as such studies are essential to ensure the safety of the compound in therapeutic applications. Moreover, while this study demonstrated significant biochemical, histological, and microstructural changes associated with NAC administration, it did not directly evaluate the molecular pathways involved in these effects. Future studies incorporating molecular or immunohistochemical analyses, such as the evaluation of inflammatory cytokines, osteoclast activity, or RANKL/OPG signaling, would be important to further elucidate the mechanisms of the protective effects of NAC in periodontal tissues.

## Conclusions 

The systemic administration of N-acetylcysteine demonstrated promising therapeutic potential in addressing the pathological changes associated with periodontitis. Our results indicate that N-acetylcysteine influenced the oxidative biochemistry of the gingival tissue, enhancing endogenous antioxidant defense. Histopathological analysis revealed a marked reduction in inflammatory infiltration and preservation of essential cellular components. Additionally, collagen content analysis highlighted N-acetylcysteine ability to protect against the degradation of collagen fibers, maintaining the integrity of the extracellular matrix. Micro-computed tomographic analysis further supported these findings, demonstrating preservation of bone quality parameters and a significant reduction in vertical alveolar bone loss. These findings position N-acetylcysteine as a valuable candidate for further exploration in the context of periodontal therapy, emphasizing the need for additional studies to confirm its efficacy and establish its clinical applicability.

## Supplementary Information

Below is the link to the electronic supplementary material.

ESM 1 (DOCX 24.5 KB)

## Data Availability

All data generated or analyzed during this study are included within this article. Additional information can be provided upon reasonable request from the authors.
