# Supplementary material for: N-acetylcysteine mitigates oxidative stress and reduces alveolar bone damage on experimental periodontitis in rats
Source: Naunyn Schmiedebergs Arch Pharmacol. 2026 May 14;399(11):16963–78. doi: 10.1007/s00210-026-05421-7 (PMC13503443; doi:10.1007/s00210-026-05421-7)
Supplement: Supplementary file 1 [file 210_2026_5421_MOESM1_ESM.docx]

**N-Acetylcysteine Mitigates Oxidative Stress and Reduces Alveolar Bone Damage on Experimental Periodontitis in Rats**

**Periodic:** Naunyn-Schmiedeberg's Archives of Pharmacology

Ana Paula de Andrade Silva ^1^, Erick Alves dos Santos ^1^, Deborah Ribeiro Frazão^1^, Vinicius Ruan Neves dos Santos ^1^, José Mario Matos-Sousa ^1^, Beatriz Risuenho Peinado^1^, Deiweson Souza-Monteiro^1^, Wallacy Watson Pereira Melo ^1^, Zuleni Alexandre da Silva ^1^, Everton Luiz Pompeu Varela ^2^, Jorddy Neves da Cruz ^1^, Carina Maciel da Silva-Boghossian^3^, Fabrício Mezzomo Collares^4^, Renata Duarte de Souza-Rodrigues ^1^, Rafael Rodrigues Lima ^1*^.

^1^Laboratory of Functional and Structural Biology, Institute of Biological Sciences, Federal University of Pará, Belém 66075-110, PA, Brazil.

^2^Oxidative Stress Research Laboratory (LAPEO), Institute of Biological Sciences (ICB), Federal University of Pará (UFPA), Belém 66075-110, PA, Brazil

^3^ Dental Clinic Departament, Universidade Federal do Rio de Janeiro, Rio de Janeiro 21941-913, RJ, Brazil.

^4^Dental Materials Laboratory, Department of Conservative Dentistry, School of Dentistry, Federal University of Rio Grande do Sul, Porto Alegre 90010-150, RS, Brazil.

* **Correspondence:**

Rafael Rodrigues Lima, Ph.D.

Laboratory of Functional and Structural Biology, Institute of Biological Sciences,

Federal University of Pará, Augusto Corrêa street nº 1, Campus do Guamá, Belém,

Pará, Brazil. 66075-110. E-mail address: [rafalima@ufpa.br](mailto:rafalima@ufpa.br)

**Supplementary material 1:**

**Arrive Guidelines Table**

**ARRIVE Guidelines 2.0: Author Checklist**

**The ARRIVE Essential 10**

| Item | Recommendation | Section/line number, or reason for not reporting |
| --- | --- | --- |
| 1. Study design | For each experiment, provide brief details of study design including: a. The groups being compared, including control groups. If no control group has been used, the rationale should be stated. b. The experimental unit (e.g., a single animal, litter, or cage of animals). | Section 2.1 (Experimental groups): The study design is clearly described, with three groups: control, experimental periodontitis (EP), and EP + N-acetylcysteine (NAC). The experimental unit is a single animal (rat). |
| 2. Sample size | a. Specify the exact number of experimental units allocated to each group, and the total number in each experiment. Also indicate the total number of animals used. b. Explain how the sample size was decided. Provide details of any a priori sample size calculation, if done. | Section 2.1 (Experimental groups): The sample size is specified as 8 rats per group (total of 24 rats). The sample size calculation was performed using G*Power software. |
| 3. Inclusion and exclusion criteria | a. Describe any criteria used for including and excluding animals (or experimental units) during the experiment, and data points during the analysis. Specify if these criteria were established a priori. If no criteria were set, state this explicitly. b. For each experimental group, report any animals, experimental units, or data points not included in the analysis and explain why. If there were no exclusions, state so. c. For each analysis, report the exact value of n in each experimental group. | Section 2.2 (Inclusion and Exclusion Criteria): Inclusion criteria are described (healthy animals with no signs of prior disease). No exclusions were made, and all animals in the periodontitis group had the lesion successfully induced. |
| 4. RandomiZation | a. State whether randomization was used to allocate experimental units to control and treatment groups. If done, provide the method used to generate the randomization sequence.  b. Describe the strategy used to minimize potential confounders such as the order of treatments and measurements, or animal/cage location. If confounders were not controlled, state this explicitly. | Section 2.1 (Experimental groups): Randomization was performed using a random number generator (<https://www.random.org/>). The procedure was conducted by two authors, but blinding during randomization was not possible due to their involvement in the experimental setup. |
| 5. Blinding | Describe who was aware of the group allocation at the different stages of the experiment (during the allocation, the conduct of the experiment, the outcome assessment, and the data analysis). | Section 2.6 (Euthanasia and sample collection): Researchers performing histological, biochemical, and micro-CT analyses were blinded to group assignments. But in Section 2.1 (Experimental groups), the authors state that they were not blinded during the randomization process and were aware of group allocations. |
| 6. Outcome measures | a. Clearly define all outcome measures assessed (e.g., cell death, molecular markers, or behavioural changes). b. For hypothesis-testing studies, specify the primary outcome measure, i.e., the outcome measure that was used to determine the sample size. | Section 2.6 Euthanasia and sample collection: Outcome measures are clearly defined, including antioxidant capacity (ABTS•+ and DPPH• assays), lipid peroxidation (LPO), histopathological analysis, collagen content, and micro-CT analysis of bone quality. The primary outcome measure is bone loss. |
| 7. Statistical methods | a. Provide details of the statistical methods used for each analysis, including software used. b. Describe any methods used to assess whether the data met the assumptions of the statistical approach, and what was done if the assumptions were not met. | Section 2.10 (Statistical analysis): Statistical methods are described, including Shapiro-Wilk test ANOVA, MANOVA, and Pearson’s correlation. Software used (GraphPad Prism, Jamovi) is mentioned. |
| 8. Experimental animals | a. Provide species-appropriate details of the animals used, including species, strain and substrain, sex, age or developmental stage, and, if relevant, weight. b. Provide further relevant information on the provenance of animals, health/immune status, genetic modification status, genotype, and any previous procedures. | Section 2.1 (Experimental groups): Species (Rattus norvegicus, Wistar), sex (male), age (90 days), and weight (180-200 g) are provided. In addition, Section 2.2 (2.2 Inclusion and Exclusion Criteria) states that all animals were healthy. |
| 9. Experimental procedures | For each experimental group, including controls, describe the procedures in enough detail to allow others to replicate them, including: a. What was done, how it was done and what was used. b. When and how often. c. Where (including detail of any acclimatisation periods). d. Why (provide rationale for procedures). | Section 2.3 (Induction of periodontitis by ligature): Procedures are described in detail, including the induction of periodontitis, NAC supplementation, and euthanasia. |
| 10. Results | For each experiment conducted, including independent replications, report: a. Summary/descriptive statistics for each experimental group, with a measure of variability where applicable (e.g., mean and SD, or median and range). b. If applicable, the effect size with a confidence interval. | Section 3 (Results): Results are reported with summary statistics (mean ± SD) for each group. |

**The Recommended Set**

| Item | Recommendation | Section/line number, or reason for not reporting |
| --- | --- | --- |
| 11. Abstract | Provide an accurate summary of the research objectives, animal species, strain and sex, key methods, principal findings, and study conclusions. | Abstract (Page 2 and 3): The abstract provides a clear summary of objectives, methods, results, and conclusions. |
| 12. Background | a. Include sufficient scientific background to understand the rationale and context for the study, and explain the experimental approach. b. Explain how the animal species and model used address the scientific objectives and, where appropriate, the relevance to human biology. | Section 1 (Introduction): The background provides sufficient context for the study, including the role of oxidative stress in periodontitis and the rationale for using NAC. |
| 13. Objectives | Clearly describe the research question, research objectives, and, where appropriate, specific hypotheses being tested. | Section 1 (Introduction): The research question and objectives are clearly stated. |
| 14. Ethical statement | Provide the name of the ethical review committee or equivalent that has approved the use of animals in this study, and any relevant license or protocol numbers (if applicable). If ethical approval was not sought or granted, provide a justification. | Section 2.1 (Experimental groups): Ethical approval is mentioned (CEUA no. 3160221220, Federal University of Pará). |
| 15. Housing and husbandry | Provide details of housing and husbandry conditions, including any environmental enrichment. | Section 2.1 (Experimental groups): Housing conditions are described, including cage dimensions, food, water, light/dark cycle, temperature, and relative humidity. |
| 16. Animal care and monitoring | a. Describe any interventions or steps taken in the experimental protocols to reduce pain, suffering and distress. b. Report any expected or unexpected adverse events. c. Describe the humane endpoints established for the study, the signs that were monitored and the frequency of monitoring. If the study did not have humane endpoints, state this. | Section 2.3 (Induction of periodontitis by ligature) and Section 2.6 (Euthanasia and sample collection): Measures to minimize pain (anesthesia) and humane endpoints (e.g., severe weight loss, lethargy) are described. No adverse events were observed. |
| 17. Interpretation/ scientific implications | a. Interpret the results, taking into account the study objectives and hypotheses, current theory and other relevant studies in the literature. b. Comment on the study limitations including potential sources of bias, limitations of the animal model, and imprecision associated with the results. | Section 4 (Discussion): Results are interpreted in the context of the study objectives and literature. Limitations are mentioned. |
| 18. Generalisability/ translation | Comment on whether, and how, the findings of this study are likely to generalise to other species or experimental conditions, including any relevance to human biology (where appropriate). | Discussion: The relevance to human biology is briefly mentioned. |
| 19. Protocol registration | Provide a statement indicating whether a protocol (including the research question, key design features, and analysis plan) was prepared before the study, and if and where this protocol was registered. | No protocol was previously made. |
| 20. Data access | Provide a statement describing if and where study data are available. | Page 25 (Data Availability Statement): Data availability is mentioned, with additional information available upon request. |
| 21. Declaration of interests | a. Declare any potential conflicts of interest, including financial and non-financial. If none exist, this should be stated. b. List all funding sources (including grant identifier) and the role of the funder(s) in the design, analysis and reporting of the study. | Page 24 (Declaration of competing interests): No conflicts of interest are declared. In section 10, funding sources are explicitly listed. |
